# Volatile codes: Correlation of olfactory signals and reception in *Drosophila*-yeast chemical communication

**DOI:** 10.1038/srep14059

**Published:** 2015-09-22

**Authors:** Nicole H. Scheidler, Cheng Liu, Kelly A. Hamby, Frank G. Zalom, Zainulabeuddin Syed

**Affiliations:** 1 Department of Biological Sciences & Eck Institute for Global Health University of Notre Dame, Notre Dame, IN 46556, USA; 2 Center for Research Computing, University of Notre Dame, Notre Dame, IN 46556, USA; 3 Entomology Department, University of Maryland, College Park, MD 20742, USA; 4 Entomology and Nematology Department, University of California, Davis, CA 95616, USA

## Abstract

*Drosophila* have evolved strong mutualistic associations with yeast communities that best support their growth and survival, resulting in the development of novel niches. It has been suggested that flies recognize their cognate yeasts primarily based on the rich repertoire of volatile organic compounds (VOCs) derived from the yeasts. Thus, it remained an exciting avenue to study whether fly spp. detect and discriminate yeast strains based on odor alone, and if so, how such resolution is achieved by the olfactory system in flies. We used two fly species known to exploit different niches and harboring different yeasts, *D. suzukii* (a pest of fresh fruit) and *D. melanogaster* (a saprophytic fly and a neurogenetic model organism). We initially established the behavioral preference of both fly species to six *Drosophila-*associated yeasts; then chemically analyzed the VOC profile of each yeast which revealed quantitative and qualitative differences; and finally isolated and identified the physiologically active constituents from yeast VOCs for each drosophilid that potentially define attraction. By employing chemical, behavioral, and electrophysiological analyses, we provide a comprehensive portrait of the olfactory neuroethological correlates underlying fly-yeast coadaptation in two drosophilids with distinct habitats.

In insects, as in other animals, senses are biological features that have been shaped by natural selection to promote adaptive behaviors^1^,^2^,^3^. Among these, olfaction is pronounced^4^,^5^. As insects adapt to different ecological environments and specialize on novel hosts, typified by unique chemical landscapes, their olfactory structures and molecular correlates diverge and adapt enabling them to locate suitable hosts, habitats, oviposition sites and conspecifics^4^,^6^. A thorough understanding of this evolutionary process and interactions can be exploited to identify baits unique to a pest species that can be used as safe and effective means of pest monitoring and management. The development of a bait for *Rhagoletis pomonella*, a pest of cultivated apples and model for incipient sympatric speciation, demonstrates the use of these principles^7^: *R. pomonella* was shown to be attracted to a volatile chemical blend derived from its introduced host, apple, over the ancestral host plant, hawthorn. In insects, a distinct and limited range of volatiles from diverse sources are parsimoniously used in various contexts eliciting strong olfactory behaviors^8^. These volatile chemicals are largely detected and discriminated by odorant receptors (ORs), a divergent family of proteins, that are expressed in olfactory receptor neurons (ORNs) located inside hair-like structures, sensilla, on the antennae and other olfactory organs^9^.

*Drosophila melanogaster* (Diptera: Drosophilidae), one of the most intensely studied organisms, has been at the forefront of seminal discoveries, many involving olfaction, for over four decades^10^. Unfortunately, this extensive knowledge derived from *Drosophila* studies has not been transferred to applied entomological problems because *Drosophila* have rarely been considered direct pests of economic importance. However, the recent introduction of a highly pestiferous east Asian vinegar fly, *Drosophila suzukii* (Matsumura) (Diptera: Drosophilidae), into mainland North America in the late 2000 s, and an increasing presence of this pest throughout Europe, has changed that perspective^11^. While the vast majority of over 1500 known species of *Drosophila*^12^ have typically been considered innocuous or simply a nuisance as they feed and oviposit on damaged, decaying, or fermenting fruits, *D. suzukii* is one of the few species that has evolved a serrated ovipositor^13^ enabling them to pierce the skin of fresh fruits and lay their eggs inside the flesh. Reductions in yield of berry and soft fruit crops in newly invaded areas of North America and Europe are reported to reach as high as 80% in the absence of any management practices, although a current and comprehensive economic assessment is lacking^11^,^14^.

In order to study the olfactory sensory correlates that help guide flies to and define a preferred substrate, a sound understanding of the hosts and their derived odors is critical. Yeasts constitute the main nutritional source for adults and larvae of most *Drosophila* species^15^, and the nutritional value of yeast-enriched habitats is advertised by a rich volatile chemical landscape^16^ which mediates critical life traits such as host preference^16^,^17^, mate location and oviposition^18^. In addition to detecting yeasts, flies also discriminate among yeast species. They appear to prefer those yeasts that enhance critical life history traits^15^,^19^,^20^,^21^. A screening of 43 yeast isolates against *D. melanogaster* in a behavioral choice assay identified acute olfactory preference for strongly-fermenting, fruit associated yeasts^20^. One of the best studied models for fly-yeast coadaptation and coevolution has been the case of *D. mojavensis* flies and their host cacti with their unique yeast populations in the Sonoran desert^21^,^22^. In a detailed behavioral analysis involving multiple fly and yeast species, Dobzhansky *et al.*^17^ demonstrated species-specific preferences of flies to the yeast from their own host/habitats. Recent investigations have shed new light into the chemosensory basis of this association between four *D. mojavensis* populations (incipient species) and their host cacti^23^, establishing a strong correlation between the volatile chemistry of the hosts (cacti) and the olfactory preference in populations of *D. mojavensis*. One population specialized on the barrel cactus, *Ferocactus cylindraceus*, has significantly reduced antennal sensitivity to esters and increased responses to aromatic compounds that match the relative decrease in esters and increase in the aromatic compounds in the barrel cactus volatile profile. This study provided compelling evidence for alteration at the periphery to adapt to different host plants based on volatile chemosensory cues. It is still an open debate if the adaptive changes in olfaction predate host specialization or occur as a consequence, as argued for other fly species^24^.

We therefore conducted a comprehensive investigation to understand the factors that potentially facilitate *D. suzukii* flies in exploiting novel food substrates, with an overarching aim to develop effective baits that can specifically capture *D. suzukii* utilizing odor-baited traps. Recent studies towards this have identified volatiles derived from vinegar (which is commonly used as a bait against vinegar flies)^25^, foliage^26^, and fruits^27^,^28^ that could potentially attract *D. suzukii*. However, given the ubiquitous role of yeast in multiple life-traits, including their implicit role in defining *Drosophila* attraction to various substrates^18^, we focused on yeast derived volatiles as a potential source of attractants. We earlier reported the isolation and identification of certain yeast species, namely *Hanseniaspora uvarum, Pichia kluyveri*, and *Pichia terricola*, which were enriched in the alimentary canal of field collected *D. suzukii* indicating a mutually beneficial association between the fly and these yeasts^29^. A parallel study reported the favorable association of *Candida californica, Candida zemplinina*, and *Pichia kluyveri* with *D. melanogaster*^30^. There is also evidence, however, of *H. uvarum* occurring as one of the most frequent yeasts in the gut of multiple *Drosophila* spp., including *D. melanogaster*^31^. In this study, by including these two fly species and their commonly associated yeasts, we demonstrate: i) a clear behavioral preference in each fly among and between the yeasts, ii) unique yeast volatile chemical signatures that could be discretely sorted into chemical odor space, and iii) that this chemical separation is resolved into chemosensory space by the flies’ peripheral olfaction. Our findings contribute to and extend the understanding of coevolution in sensory communication^2^ between yeast and vinegar flies^23^,^32^,^33^,^34^,^35^. Our results open a promising avenue for the development of robust, species specific odor baits and contribute important insights to the understanding of the neuroethological trait evolution in two distinct and divergent drosophilids, one a highly invasive pestiferous fly while the other simply innocuous.

## Results

### Yeasts derived from field collected fly populations induce distinct preferences

**Figure 1:**
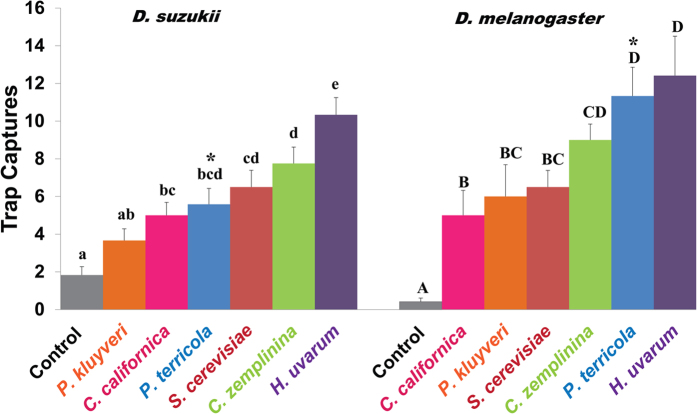
*D. suzukii* and *D. melanogaster* differentially discriminate between six yeast species based on the odors. Relative yeast preference was evaluated by simultaneously exposing flies in an arena to six traps baited with a different yeast species (represented by different colors) or PDB as control. Separate trials were carried out for each fly species with N = 12. Error bars are standard error of the means. Letters above indicate the statistical significance (*p *< 0.05; one-way ANOVA) within a fly species (indicated by small or capital letters). When preference between the two fly species for a given yeast was compared by an unpaired student’s t-test, the responses were not significantly different (*p *> 0.05) except for *P. terricola* (indicated by an asterisk).

All six yeast species (*P. terricola, P. kluyveri, H. uvarum, C. californica, C. zemplinina*, and *S. cerevisiae*), commonly associated with either of the fly species, elicited strong attraction of *D. melanogaster* and *D. suzukii* to yeast baited traps in a binary choice assay. Regardless of yeast or fly species, yeast baited traps were consistently more attractive than control traps (*p *< 0.02). The equal distribution of flies when both the traps were treated with the control bait, potato dextrose broth (PDB), demomsrated that there was no positional bias (*p *> 0.2) (Table 1). Further investigation into the relative yeast preference of each fly species under a multi-choice paradigm revealed distinct patterns. While *D. melanogaster* showed a significant discrimination among the yeasts (*F*_6,77 _= 9.12; *p *= 1.56 × 10^−7^), the statistical difference is much more robust in *D. suzukii* (*F*_6,77 _= 12.85; *p *= 5.30 × 10^−10^). Pairwise comparisons of captures for each yeast within *D. melanogaster* revealed the most significant attraction to *H. uvarum* and *P. terricola* (Fig. 1), whereas *H. uvarum* alone induced the highest attraction within *D. suzukii.* We also note with interest that a two-way ANOVA, taking yeast and fly species as factors, revealed significant difference in the overall yeast preference by two fly spp. (*F*_1,132 _= 7.78; *p *= 0.006), despite a relatively conserved preference pattern. Finally, we asked if an unpaired comparison between the two fly species for a given yeast would reveal any differences in the behavioral preference. Difference in preference was non-significant, except for *P. terricola*, which elicited a significantly higher response in *D. melanogaster* compared to *D. suzukii* (*p *< 0.005).

### Yeasts produce unique odor profiles

**Figure 2:**
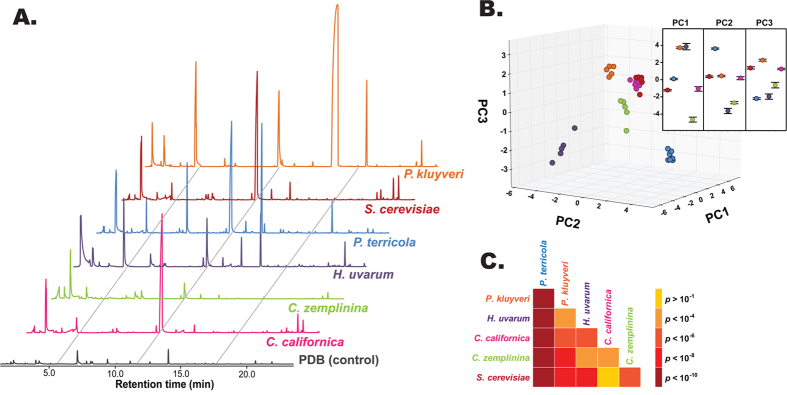
Yeast species produce distinct odor profiles. (**A**) Representative odor profiles as measured by gas chromatogram-mass spectrometry (GC-MS). Profiles are offset for clarity. (**B**) Three dimensional cluster plot based on the PCA resolves yeasts into discrete clusters. The area under the 10 most abundant chemical constituents from each yeast species was extracted; since the abundant peaks were not the same in each yeast, extracting the top 10 and retrieving them in the rest yielded a list of 24 constituents overall that were subjected to PCA. The cluster plot was generated using the first three principal components (PC1, PC2, PC3 each explained 34.1%, 24.5% and 13.6% of the total variation, respectively). The insets represent individual mean PC score ± SEM. (**C**) A heat map based on the hypothesis tests between PCA clusters indicates highly significant differences in the yeast VOC chemistry.

Analysis of the collected Volatile Organic Compounds (VOCs) from all the yeast species revealed unique profiles (Fig. 2a; Supplementary Figs S1 and S2; Supplementary Tables S1 and S2). To determine if this variation in VOCs can be utilized to resolve yeast species into distinct entities, we subjected the data to Principal Component Analysis (PCA). The area under the ten most abundant constituent odorants extracted from the Total Ion Chromatograms (TICs) from each yeast species sorted the populations into discrete clusters (Fig. 2b). More than 72% of the observed variation in the profiles was explained by the first three principal components (PCs). To identify the most influential constituent odorants (loading factors) separating yeasts, we took a loading value above 0.28 as indicative of significant contribution towards the determination of each PC. PC1, which accounted for 34.1% of the overall variation, was weighed positively by all the ester compounds except one aromatic aldehyde, benzaldehyde, whereas alcohols and ketones contributed negatively. The top positive contributors include isoamyl propionate and isoamyl acetate while 1-pentanol, 2-methyl-1-butanol, and 6-methyl-5-heptene-2-one contributed negatively, in decreasing order of impact. The next highest variation (>24%) was accounted for by PC2, which was positively impacted by ethyl octanoate, ethyl hexanoate, ethyl decanoate, and 3-(methylthio)-1-propanol, while negatively influenced by ethyl acetate and benzaldehyde. PC3 (explaining 13.6% of the total variation) was influenced positively by an aromatic, phenethyl propionate, whereas negatively contributing compounds were 3-(methylthio)-1-propanol and four esters, namely ethyl octanoate, ethyl hexanoate, ethyl decanoate and ethyl acetate (in decreasing value). Finally, the statistical significance of the differences among yeast profiles was determined by pairwise MANOVA tests of the first three PCs (Fig. 2c). Except for *C. californica* and *S. cerevisiae*, all species were significantly different from each other (*p *= 0.0001), with *P. terricola* being the most significant.

### Peripheral response repertoire resolves yeast species into distinct odor space

**Figure 3:**
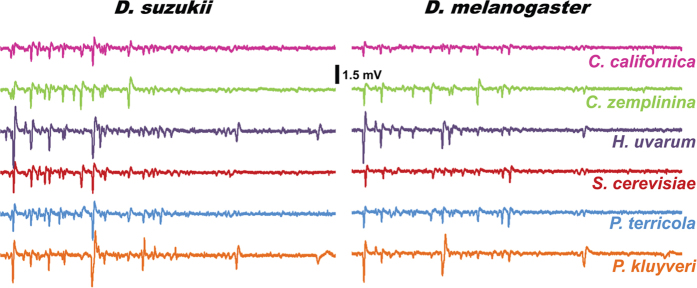
Olfactory responses to yeast derived volatiles are qualitatively and quantitatively different across yeast and fly species as measured by antennal sensitivity. Representative antennal response profiles were generated by gas chromatography linked electro-antennographic detection (GC-EAD) method which measures response to odor constituents as they elute from the GC column. Refer to Supplementary Figure S3 for the accompanying GC-FID traces; and Supplementary Figure S4 represents comparative responses.

**Figure 4:**
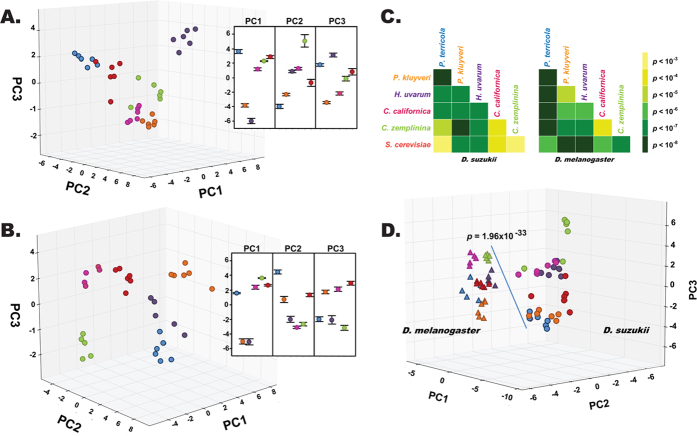
Fly antennae resolve yeast volatile headspace into discrete odor space. Principal Component Analysis (PCA) resolved antennal responses to each yeast into discrete species clusters for *D. suzukii* (**A**) and *D. melanogaster* (**B**). The 25 most intense responses induced by each yeast from both fly species were extracted. Since these responses were not the same for each profile, extracting the top 25 responses and retrieving those in the other profiles yielded a list of 53 responses overall that were subjected to PCA. Insets are as described in Fig. 2. (**C**) A heat map based on the hypothesis tests between PCA clusters indicates highly significant differences in the fly odor space. Note the inter-species difference in the resolution: while *C. zemplinina* and *S. cerevisiae* are least resolved in *D. suzukii*, the reverse is true for *D. melanogaster*. (**D**) Odor space in two fly species can be resolved by PCA.

Having established the strong preference of vinegar flies to yeast and their ability to discriminate among them, we investigated the sensory physiological basis of this attraction by using gas chromatography link electroantennographic detection (GC-EAD). Many of the yeast headspace odor constituents elicited electrophysiological responses of various intensities (Fig. 3; Supplementary Figs S3 and S4; Supplementary Tables S1 and S2). To investigate whether *D. suzukii* and *D. melanogaster* can separate yeasts into discrete odor space based on the volatile bouquet, and if this resolution is conserved between the species, we performed principal component analysis (PCA) on the 25 most intense responses compiled from each yeast response profile (Supplementary Table S1) and plotted them three dimensionally. This revealed a distinct sorting of the yeasts by the flies (Fig. 4a,b).

The first three PCs together accounted for 56.6% and 58.1% of the total variation observed in *D. suzukii* and *D. melanogaster*, respectively. The significant contribution of these three PCs in resolving six yeasts into discrete points are evidenced in the insets of Fig. 4a,b. In order to distinguish which odorants (loading factors) are most influential in fly discrimination among yeasts, we selected the three positive and negative factors of greatest impact on the determination of each PC.

For *D. suzukii* (Fig. 4a), the positively contributing constituents were as follows: PC1 (explaining 26.8% of total variation) by compound 27, ethyl butyrate, and ethyl isobutyrate; PC2 (18.5%) by compounds 3, 21, and butyl acetate; and PC3 (10.8%) by 2, 5-dimethylpyrazine, 2-heptanone and compound 49. Constituents that weighted negatively were: PC1 by compounds 1, 29, and 2-phenethyl acetate; PC2 by isoamyl acetate, compound 23, and isoamyl propionate; and PC3 by compounds 52, 30, and 43. In *D. melanogaster* (Fig. 4b), compounds which weighted positively were: PC1 (accounting for 28.7% of variation), ethyl butyrate, compound 43, and ethyl isovalerate; PC2 (16.2%), ethyl hexanoate and compounds 30 and 16; and PC3 (13.2%), compounds 40, 37, and 6. The following compounds weighted negatively: PC1 by ethyl acetate, isoamyl acetate, and compound 29; PC2 by ethyl propionate, furfuryl acetate, and butyl acetate; and PC3 by compounds 32, 7, and 25. Interestingly, PC1 in both the species is positively impacted by ethyl butyrate while negatively impacted by compound 29. Additionally, butyl acetate contributed significantly to PC2 for both fly species, however, with opposite impact. A full list of compounds with their unique identification numbers along with Kovat’s Indices (KIs) and CAS numbers is given in Supplementary Tables S1 and S2.

In order to determine the statistical significance of the odor separation represented in the PCA clustering (Fig. 4a,b), pairwise MANOVA tests were performed between group responses. Responses elicited by all six yeast species were significantly different from each other in a pairwise comparison performed on the two fly species (Fig. 4c). In *D. suzukii*, the highest difference (*p *= 2.9 × 10^−8^) was found between *P. kluyveri* vs *P. terricola* whereas the lowest difference was between *S. cerevisiae* and *P. terricola* (*p *= 0.0053). In *D. melanogaster* the highest difference was between *S. cerevisiae* and *P. kluyveri* (*p *= 1.1 × 10^−8^) and the lowest (*p *= 3.4 × 10^−8^) was between *C. zemplinina* and *C. californica*. The resolution pattern and the magnitude of separation among the yeast species were different in *D. melanogaster* compared to *D. suzukii*. We note with great interest that *D. suzukii* could be effectively separated from *D. melanogaster* (*p *= 1.96 × 10^−33^) by subjecting yeast VOC induced responses from both fly species to a single PCA (Fig. 4d). The different chemosensory space of the two fly species suggests that they can resolve complex chemical landscapes as distinct entities.

**Figure 5:**
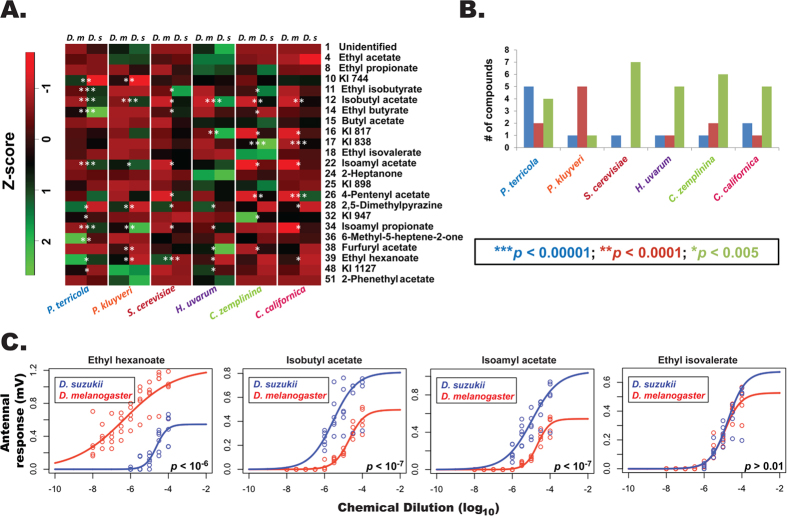
*D. suzukii* and *D. melanogaster* respond to the most effective yeast constituents with differential sensitivity and selectivity. (**A**) Heat map indicates the varying intensity of responses to an identified odor constituent (rows) in two fly species (individual columns) for a given yeast (grouped column). Antennal responses were mean averaged across a row and set to zero (Z-score; dark represents 0); higher responses (above the average) are indicated in green, whereas red indicates lower responses. White asterisks denote statistical significance between fly species across yeasts. Kovat’s Indices (KI) are used to denote compounds that could not be positively identified. (**B**) Distribution histogram representing the number of compounds eliciting significant responses between two fly species from a given yeast. (**C**) Dose-dependent electrophysiological responses measured from fly antennae upon stimulation with increasing doses of biologically active constituents identified from yeast odors that elicited significantly different responses between *D. melanogaster* and *D. suzukii* from at least five of the six yeast odor profiles. Ethyl isovalerate served as control.

Finally, to explore if the distinctly resolved sensory spaces for two species can be attributed to a set of compounds, we examined the top ten antennal responses generated by each yeast from *D. melanogaster* and *D. suzukii*, which resulted in a subset of constituents eliciting significantly different responses between the two fly species (Fig. 5a,b). Of these, a majority were esters with the exception of two ketones (2-heptanone and 6-methyl-5-heptene-2-one). Responses induced by isobutyl acetate, isoamyl acetate and ethyl hexanoate were significantly different between *D. melanogaster* and *D. suzukii* in at least five of the six yeast odor profiles suggesting the salience of these odorants contributing to behavioral discrimination. These compounds were present in varying amounts (ranging from below the detection threshold of MS up to 100 ng) in the natural headspace of each yeast profile, yet were consistently active on the antennae (Supplementary Fig. S4). We further evaluated the salience by stimulating antennae with known amounts of these compounds to establish relative sensitivity. As a control, we used ethyl isovalerate, which elicited comparable antennal responses from the two fly species for each of the yeasts tested (Fig. 5a). All four compounds elicited electrophysiological responses that were dose-dependent in both species. As expected, the dose-response curves for the test compounds were significantly different between fly species (*p *< 1.2 × 10^−7^), whereas ethyl isovalerate (control) induced comparable depolarization (Fig. 5c). Additionally, while the straight chain ester, ethyl hexanoate, elicited stronger responses (lower threshold) in *D. melanogaster*, the two other test compounds with a branched alkyl group induced responses with lower threshold in *D. suzukii*. This species-specific sensitivity confirms the critical role of a limited number of compounds potentially contributing to the behavioral differentiation.

## Discussion

Yeasts coadapt with each other in their natural habitat to form stable communities, and these communities are further coadapted with *Drosophila* resulting in mutualistic relationships^21^,^23^. *Drosophila* thus appear to have evolved a strong preference for those yeasts which best support their growth and survival, as measured by various fitness traits^15^,^19^,^21^. These interactions between yeasts and flies can be directly correlated with the odor induced preference in flies to their coadapted yeasts^17^,^36^. Furthermore, this mutualistic association is conserved across large taxonomic groups, and the evolution of preference between microorganisms and insects has been shown to be significantly modulated by way of microbial volatile organic compounds (MVOCS)^16^.

Here, we systematically searched for the olfactory correlates of the long described fly-yeast interactions by studying two different fly species, namely *D. melanogaster* and *D. suzukii*. While the former species laid the foundation as a model species for neurogenetics, the latter species is emerging as a national threat to food security in the USA^14^. We began our investigation by collecting *D. suzukii* flies from the field and isolating and identifying the yeast species that were highly enriched in fly alimentary canals. We determined fly preference to those yeasts, both in a binary and multichoice paradigm. Of the six isolated yeasts, *H. uvarum* induced the highest trap captures under both paradigms (Table 1 and Fig. 1). Not surprisingly, *H. uvarum* was detected in the highest number of field collected *D. suzukii* flies^29^, suggesting a strong coadaptation between these two organisms. Of note, a large scale field sampling of *Drosophila* spp. and their associated microbes (yeasts and bacteria) identified *H. uvarum* group members as one of the most frequent yeast groups from *D. melanogaster* flies as well^31^.

Signals and reception rarely, if ever, arise *de novo*, and this has been elegantly demonstrated to evolve in synchrony in insect chemical communication, for which the term “coevolution” was first coined^37^,^38^,^39^. Fly-yeast interactions, as mediated by the rich yeast volatile signal and fly OR repertoires have been suggested as a powerful tool to study ecological interactions and coadaptation^32^. We used a two pronged approach to characterize the chemosensory correlates defining fly-yeast associations, encompassing yeast chemical analysis (*signals*) and measuring fly olfactory sensitivity and selectivity (*reception*). Our chemical analyses revealed species-specific odor signatures that could be separated with high statistical significance between all but *C. zemplinina* in comparison to *S. cerevisiae* (Fig. 2; Supplementary Figs S1 and S2). A recent study involving 14 diverse *S. cerevisiae* accessions of known genetic background also found that distinct yeast populations could be resolved solely based on volatile constituents^32^. Furthermore, it correlated the major constituents impacting principal components with chemosensory receptor repertoires (ORs and IRs), suggesting them as potential determinants in fly-yeast coevolution. Our meticulous measurements of yeast VOCs are largely in agreement with two other extensive studies on yeasts volatile chemistry of *S. cerevisiae*, one which analyzed VOCs on polar and non-polar columns^32^, and another which analyzed VOCs produced by yeasts raised on different substrates^40^. In a detailed preliminary analysis we compared and contrasted the VOC profiles from yeast that were either raised on PDB or a synthetic minimal media every 24 hrs. up to 72 hrs. Our results indicated a robust and consistent VOC profile from PDB raised yeast that did not vary significantly between the three time points (data not shown).

We extended our study to identify the olfactory-physiological correlates that potentially encoded the yeast odor separation into chemosensory space, resulting in the behavioral discrimination noted above. Olfactory response patterns from *D. melanogaster* (Fig. 4b,c) reflected the pattern of separation in the yeast chemistry, demonstrating highly significant differences for *C. zemplinina* vs. *P. terricola* as well as *H. uvarum* vs. *P. terricola.* In *D. suzukii* the observations in the chemistry patterns were only partially preserved (Fig. 4a,c). Taken together, these patterns indicate that the chemical differences in the yeast VOC profiles (as described by our PCA) are translated into complex behavioral output, though not comparable between fly species (Table 1 and Fig. 1). This can further be illustrated by the pairwise z-score comparisons (after centered Gaussian normalization) of the top electrophysiological response inducing compounds between two fly species (Fig. 5a,b) wherein esters represented the largest group. Esters are mainly formed via two distinct pathways in yeasts, one resulting in acetate esters (AE) and the other in ethyl esters (EE)^41^. A large screening of 38 yeast strains belonging to 5 genera revealed yeast of the *Pichia* and *Hanseniaspora* genera as the best producers of acetate esters^42^. Recent studies additionally demonstrated how certain yeasts strains employ the AE biosynthetic pathway for their active dispersal through flies, and those yeasts are found in highest abundance from field caught flies^43^. Our analyses corroborate those findings in the case of *D. melanogaster* wherein, *Hanseniaspora* and *Pichia* elicited significantly higher trap captures under both the regimes (Table 1 and Fig. 1). Responses from *D. suzukii* were more intruiging. We note with great interest that two esters of AE class (isobutyl and isoamyl acetate) induced significantly higher olfactory responses from *D. suzukii* compared to *D. melanogaster*, whereas ethyl hexanoate (EE class) had an opposite effect suggesting complex interactions (Supplementary Fig. S4). Overall, our extensive analysis highlights the relative significance of a few shared constituents (Fig. 5b,c) that potentially facilitate *D. suzukii* and *D. melanogaster* in discerning their own niche in complex yeast chemical landscapes.

Given that only a handful of compounds, the majority of which transpired to be esters, appear to encode enough information enabling two fly species to detect and discriminate their niches, it poses the question of how this is achieved. In flies, this feat is proposed to be accomplished by multiple means: an overall alteration in the OR repertoires adapted for niche specialization^44^,^45^,^46^,^47^,^48^; changes in the amino acid residues of a given OR that renders them differentially sensitive^49^; changes at the peripheral olfactory apparatus such as an altered number of specialized sensilla/ORNs^50^,^51^,^52^; and finally the modulation of the transduced signal by the interneurons, projection neurons and Kenyon cells^53^,^54^. Further, though odorants are parsimoniously used in multiple contexts^6^,^8^, odor constituents of high salience usually have lower sensory thresholds and are detected by a small number of specialized (narrowly tuned) ORs, whereas the majority of chemical signals are detected with higher threshold by a large number of broadly tuned ORs^4^,^54^. Niche utilization studies in *D. sechellia* identified methyl hexanoate as a key chemostimulant produced by its specialized host, morinda fruit. Flies responded with extremely low threshold to this ester and *D. sechellia* were found to have an increased number of sensillae/ORNs detecting hexanoates, also leading to a corresponding increase in the size of DM2 glomerulus which receives the sensory input^50^. Flies thus employ many strategies, alone and/or in concert, to decipher the chemical landscape around them by extracting quantitative and qualitative features of odors which are translated into meaningful percepts. In our study, *D. suzukii*’ s reduced sensitivity to ethyl hexanoate and significantly higher sensitivity to isobutyl acetate and isoamyl acetate as compared to *D. melanogaster* represent an exciting avenue to study the salience of these ligands at the molecular, cellular, glomerular and perceptual level in this pestiferous fly.

Environment greatly impacts the *Drosophila* yeast flora^15^. Though the precise origin of *D. suzukii* remains unknown, its historical range covers much of eastern Asia and now extends widely from East Asia, to Hawaii, North and Central America, as well as Europe. An extensive analysis of 246 individuals from 12 populations did not reveal any population diversity patterns that could contribute to reconstruct its invasion history into the western hemisphere^55^. Based on this, we can assume that *D. suzukii* have had multiple alternate hosts and habitats before their ongoing major shift to monocultured fruits. An exciting avenue for future research is to explore those ancestral and alternate habitats which can potentially offer a rich resource to isolate and identify unique yeasts and/or their derived VOCs for the development of baits towards manipulating the populations of an economically important emerging pest. Such studies would also provide novel insights into the coevolutionary history of the pestiferous and invasive *D. suzukii* flies and their associated yeasts.

## Material and Methods

### Fly Husbandry

Oregon R wild-type *Drosophila melanogaster* and the field-derived *Drosophila suzukii* WT3 line [the line used for sequencing and described in detail by Chiu *et al.*^56^] were maintained on standard cornmeal diet in 2.3 cm × 9.4 cm clear plastic fly culture vials at the laboratory temperature of 22.5 ± 0.5 °C with lights on approximately at 8:00 AM and off at approximately 8:00 PM.

### Yeast Culturing

Six yeast cultures (*Hanseniaspora uvarum, Pichia terricola, Pichia kluyveri, Candida californica, Candida zemplinina*, and *Saccharomyces cerevisiae*) were obtained from the Phaff Yeast Collection at University of California-Davis from Dr. Kyria Boundy-Mills. These yeasts were maintained as stock cultures in the laboratory since 2012 on PDA prepared with 2.5% Potato Dextrose (HiMedia Laboratories) and 2% Agar (Alfa Aesar). Sealed plates were maintained in an incubator at 30 °C. Stocks were re-streaked every two weeks to maintain active cultures. Before experimentation, a single yeast colony from a stock culture plate was picked using a sterile plastic inoculation needle and transferred to a 15 mL test tube containing 5 mL liquid media [2.5% Potato Dextrose Broth (PDB)] to begin high density starter cultures. The tubes were placed on a shaker at 150 rpm at 30 °C for at least 24 hrs. that resulted in an optical density (OD) of ≥1.8 which was earlier determined to produce a representative volatile profile (data not shown) in each yeast except *C. zemplinina*, which needed 48 hrs. ODs were measured, using at least three replicates, at 600 nm absorbance on a spectrophotometer (BioPhotometer plus, Germany) with sterile PDB as the optical blank. Large volume cultures of 50 mL (≥1.8 OD) were started in 125 mL glass bottles by using 0.5 mL of inoculum from a mature starter under the same conditions. Larger cultures were aliquoted to 10 mL volumes for parallel odor extraction and physiology experiments. Starter cultures were directly used for behavioral experiments.

### Fly Behavior

Attraction to the six yeasts was tested for both the fly species under two regimes. In the first setup, attraction of each yeast species was tested against a control. In the next set of experiments, the relative attraction of all the yeasts was compared with each other.

Fly traps were designed after Syed *et al.*^57^, with minor modifications: 1.5 mL SeaLRite micro-centrifuge tubes (USA Scientific, Inc.) were cut 3 mm from the tapered end (bottom) into which a 1000 μL blue plastic pipette tip (Neptune Scientific, CA) was inserted. The pipette tips were cut 0.5 cm from the narrow end and 2.5 cm from the large end. This resulted in an inverted trap design (Supplementary Fig. S5) wherein the lid of the micro-centrifuge tube could be used to either hold 125 μL of yeast culture or an equal volume of control broth (PDB). Two to four day old flies that were starved for 20 hours on 1% agar (Alfa Aesar, MA) were used for behavioral assays.

In order to assess the attraction of individual yeasts, a trap was baited with 125 μL of yeast culture and another trap holding an equal volume of PDB served as the control. Traps were secured to the base of 266.1 mL clear conical plastic cups (4.5 cm base; 7 cm top and 11 cm height from a commercial vendor) approximately 2.5 cm apart using ~0.8 cm squares of double sided adhesive tape (3M Scotch, USA). Next, relative attraction of the six yeast species was compared by exposing flies to all yeasts at once. A 1000 mL glass beaker (10.5 cm base; 16 cm height) served as the arena. Each trap baited with a given yeast species, or control (PDB), was placed 3 cm apart and 3.5 cm from the arena center. Flies were aspirated to the behavioral arenas which were covered with two layers of grade 50 cheese cloth secured with a rubber band. Behavioral assays began approximately at 4:00 pm and ran for a 24 hr. period at 22.5 ± 0.5 °C. Each cup or beaker was considered a replicate. Flies of mixed sex and age were tested. For single yeast experiments, twenty flies were used whereas sixty flies were used for comparative attraction tests.

### Yeast Odors Analysis

A 10 mL aliquot of ≥1.8 OD yeast culture was transferred into a 20 mL disposable glass scintillation vial (Kimble, IL) and sealed with Teflon^®^ tape (Sigma-Aldrich). A gray SPME fiber [23 ga StableFlex^TM^ coated with Divinylbenzene/Carboxen/Polydimethylsiloxane (DVB/CAR/PDMS), Supelco Analytical] was cleaned as per the manufacturer’s instructions and exposed to the yeast headspace for 3 to 4 hrs. by piercing through the Teflon® seal. Exposed fibers were then used either for odor profile analysis by Gas Chromatogram-Mass Spectrometry (GC-MS) (GC: Agilent Technologies, CA. 7890A model; MS: Inert XL MSD with a triple-Axis Detector, Agilent Technologies, CA. 5975C) or Gas Chromatogram linked Electro-antennographic detection (GC-EAD) in parallel with Flame Ionization Detection (FID). We used three means by which to maximize the efficiency of our yeast VOC analytical measurements: (1) we employed a SPME for VOC collection which greatly improved the odor profiles obtained relative to previous solvent extractions, (2) we used a general purpose high resolution non-polar column (HP-5) to resolve yeast at high temperatures, and (3) used two independent means of detection, flame ionization detection and mass-spectrometry to capture more constituents. Additionally, GC-FID /EAD and GC-MS analyses were performed simultaneously for a given yeast on SPME fibers that were exposed concurrently.

#### Chemical analysis by GC-MS

Exposed SPME fibers were desorbed in a spilt-splitless injector of the GC operated under splitless mode for five minutes at 250 °C. The eluted compounds were resolved on Agilent HP-5 capillary column (30 m, 0.25 mm ID, 0.25 μm phase) using helium (Ultra High Purity 5.0 Grade; Airgas, USA) as the carrier gas at 1 mL/min constant flow. The GC program was: 50 °C for one minute, increased to 280 °C at 5 °C per minute and held for 5 minutes at the final temperature. The MS was operated at 70 eV; data recording and quantification was performed with Agilent MSD ChemStation software (E.02.02.1431). Chemical identity was determined using three methods: NIST 2011 MS library, comparison of Kovat’s Retention Indices, and finally confirming biological activity with synthetic standards. Synthetic standards of highest available purity were acquired from Sigma Aldrich.

#### Isolation and Identification of biologically active yeast odor constituents by GC-EAD

Biologically active constituents from the yeast odors were isolated by SPME-GC-EAD wherein odor exposed SPME fibers were injected onto the GC and the resolved constituents were monitored, in parallel, by a FID and antennae as biological detectors. Exposed SPME fibers were processed as above on an Agilent HP-5 capillary column (30 m, 0.32 mm ID, 0.25 μm phase thickness) with a flow rate of 3 mL/min. Resolved column effluents were split 2:1 between the antenna and the FID respectively.

Female *Drosophila* were used throughout all experiments. Flies were restrained as per the original established protocol^58^,^59^ with minor modifications. The restrained fly was mounted upright on a glass slide and the electrodes used were composed of silver wire inserted into drawn-out borosilicate glass capillaries (World Precision Instruments, Inc., FL) filled with 0.1 M KCl (BDH0258-500G, BDH) saline. The reference electrode was placed in the eye of the restrained fly after which the entire preparation was moved to a high magnification microscope (Olympus BX51WI). The recording electrode was maneuvered with a MPM-10 Piezo Translator to make a firm contact on the dorsomedial antennal region. A humidified stream of charcoal filtered air was continuously passed over the fly preparation at ~0.8 cm/s from a glass tube positioned ~5 mm from the fly. Resolved odor constituents from the GC column were added into this flow. Antennal signals were captured using a high-impedance AC/DC pre-amplifier (10x), sent to an IDAC-4 box, and stored on a PC hard disk using GC-EAD 32 (v. 4.6; 2009). Hardware and software were from Syntech, Germany. The antennal signal was band pass filtered between 3 kHz and 0.1 Hz whereas the FID signal was not conditioned; both the signals were fed on to separate channels in the IDAC-4 and the digitized signal was fed onto the PC. At least three flies of each species were tested for each yeast odor with up to three recordings of the same yeast odor per fly. Recordings were performed in the afternoons and typically extended into the evening.

To generate dose-response curves, we used the GC-EAD regime (as above) instead of an offline EAG protocol to ensure comparable delivery of the odorants. The later method differentially affects the dose delivered. GC-EAD recordings were done using a mixture consisting of isobutyl acetate, isoamyl propionate, ethyl hexanoate, and ethyl isovalerate diluted decadicly relative to each compound from 10 pg to 100 ng in double distilled hexane. All the synthetic standards were ≥99.0% (Sigma Aldrich) with the exception of isoamyl acetate at ≥95% (SAFC).

### Data Processing and Statistical Analysis

*Behavior.* Percent trap captures from binary choice assay were subjected to a Mann-Whitney test. Data from the multi-choice assay for a given fly species were analyzed by one-way ANOVA and F-test. Subsequently, trap captures from the two fly species to a given yeast were analyzed by unpaired student’s t-test. The interaction between the two fly species was assessed using a two-way ANOVA.

#### GC-MS

In order to analyze the odor profiles of each yeast, the 10 most abundant peaks (by area in the Total Ion Chromatograms, TIC), were selected for further analysis. Since the top 10 compounds were not the same in all yeast odor profiles, a compilation of the top 10 from each species resulted in 24 compounds. Attempts were made to locate each of the 24 compounds in every yeast profile. Absolute areas under chosen peaks were integrated for each replicate. In order to correct for the possible variation in collection amounts among replicates, the area under all the selected peaks from a given TIC was pooled and the percent contribution of each compound was determined.

#### GC-EAD

Each yeast species was tested 5 or 6 times on each fly species. The absolute amplitude of the responses were measured (in μV) from the onset of depolarization (baseline) to the maxima of the deflection. Antennal responses elicited by the biologically active constituents from at least three of five or four of six replicates for a given species were considered reproducible. Of these, only compounds that elicited responses ≥250 μV from either fly species were chosen. The top 25 most active peaks from this data set were selected for further analysis. Since the top 25 responses were not the same to all yeast odor profiles, a compilation of the top 25 from each species resulted in 53 biologically active compounds. Attempts were made to locate each of the 53 responses in every yeast profile. In order to correct for the possible variation in antennal sensitivity between individual *D. melanogaster* or *D. suzukii* flies, each individual response was weighed against the maximum. Finally, relative sensitivity of the two fly species to the major biologically active constituents was compared (pairwise) by generating a heat map.

#### PCA

Yeast volatile profiles and the electrophysiological responses elicited by the biologically active constituents in two fly species were analyzed by PCA using percentile data. Centered Gaussian normalization was applied in order to bring the percentages of all chemicals constituents or the induced response amplitudes to the same scale. The first three principal components (together explaining more than 55% variance) were retained for subsequent statistical analysis and producing a 3-D plot for visualization. Each of the remaining principal components (PCs) accounted for a proportion of variance of single digits, and therefore was not kept. Multivariate analysis of variance (MANOVA) was performed with the first three PCs being the dependent variables. Pairwise MANOVA tests were performed between the yeast VOC profiles to identify quantifiable differences, and between the response profiles of either fly species to the biologically active yeast VOCs. The pairwise comparisons were summarized into *p*-value matrices. All statistical analyses were conducted using R3.1.1 60. Python Matplotlib1.3.1 package was used to prepare the 3D PCA plots^61^.

#### Dose-Response Function

We used a slightly modified sigmoidal function to fit the dose-response data to derive the critical parameters. The fitted model was


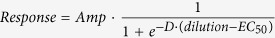


wherein *Amp* is the maximum amplitude, *D* reflects the response sensitivity as a function of dilution (steepness of the curve at dilution equaling *EC*_50_ is proportional to *D*), and *EC*_50_ represents the dilution at half maximum response. Wilcoxon signed-rank test was used to compare the significance between fly species.

## Additional Information

**How to cite this article**: Scheidler, N. H. *et al.* Volatile codes: Correlation of olfactory signals and reception in *Drosophila*-yeast chemical communication. *Sci. Rep.* **5**, 14059; doi: 10.1038/srep14059 (2015).

## Supplementary Material

Supplementary Information

## Figures and Tables

**Table 1:** Yeast derived odors induce ubiquitous preference in *D. suzukii*and *D. melanogaster*.

Yeast spp.	Percent mean trap captures ± SEM					
	*D. suzukii*			*D. melanogaster*		
	Control	Control		Control	Control	
PDB	53.14 ± 4.55	46.39 ± 4.55	ns	44.83 ± 6.49	55.17 ± 6.49	ns
	Treatment	Control		Treatment	Control	
*P. terricola*	82.41 ± 4.06	17.59 ± 4.06	***	88.32 ± 4.07	11.68 ± 4.07	***
*P. kluyveri*	78.65 ± 2.90	21.35 ± 2.90	***	73.63 ± 9.43	29.83 ± 9.43	*
*C. zemplinina*	87.24 ± 1.33	12.76 ± 1.33	***	91.40 ± 4.40	8.60 ± 4.40	***
*C. californica*	75.93 ± 3.17	24.07 ± 3.17	***	84.49 ± 5.03	15.51 ± 5.03	***
*H. uvarum*	87.53 ± 2.79	12.47 ± 2.79	***	71.47 ± 6.84	28.53 ± 6.84	**
*S. cerevisiae*	86.37 ± 3.31	13.63 ± 3.31	***	83.85 ± 4.99	16.15 ± 4.99	***

Data represents captures from the traps baited with the yeast or PDB (control) in binary choice assays. The percentages of fly captures in the treatment versus control traps in a given arena are reported for each yeast species indicating consistently significant preference for yeast in both fly species (rows). The top row (shaded; control) confirms no positional bias in fly preference in these assays. All *p-*values are calculated by Mann-Whitney test; mean ± SEM, N ≥ 9 per yeast type for each fly species.
