# Supplementary material for: Volatile codes: Correlation of olfactory signals and reception in Drosophila-yeast chemical communication
Source: Sci Rep. 2015 Sep 22;5:14059. doi: 10.1038/srep14059 (PMC4585764; doi:10.1038/srep14059)

Supplementary Information

Volatile codes: Correlation of olfactory signals and reception in *Drosophila*-yeast chemical communication

Nicole H. Scheidler[1], Cheng Liu[2], Kelly A. Hamby[3], Frank G. Zalom[4] and Zainulabeuddin Syed[1]*

[1]*Department of Biological Sciences & Eck Institute for Global Health
University of Notre Dame, Notre Dame, IN 46556, USA

[2] Center for Research Computing, University of Notre Dame, Notre Dame, IN 46556, USA [3] Entomology Department, University of Maryland, College Park, MD 20742, USA
[4] Entomology and Nematology Department, University of California, Davis, CA 95616, USA

Supplementary Table and Figure Legends

Supplementary Table S1. The 25 most intense responses (marked with corresponding colors) induced by the odor constituents from each yeast in both fly species indicated as frequency of the response occurrence. Since the top 25 responses were not the same for each yeast,

a compilation resulted in a total of 53 responses. For each profile (columns), the 28 responses not part of the initial selection were retrieved or determined not present (x). Each of the 53 were given a unique identification number (Compd#) based on time of response (RT) across yeasts (indicated by different colors) and flies (represented as *D. s* and *D. m*). Numbers indicate the frequency of a response recorded. "x" resulted either due to the absence or lower abundance (below the detection threshold of the fly) of a given yeast constituent. The gray rows represent

the top 23 constituents used in the heat map analysis. Absolute identification was based on matching Kovat's Indices (KI) between FID and MS, comparable spectra with NIST library, and finally injecting synthetic standards to confirm KI and biological activity.

Compd#	RT (min)	Kovats Indices		Identified constituents*	*P. terricola*		*P. kluyveri*		*H. uvarum*		*C. californica*		*C. zemplinina*		*S. cerevisiae*	
		FID	MS Synth.		*D. s* N=6	*D. m* N=6	*D. s* N=6	*D. m* N=6	*D. s* N=6	*D. m* N=5	*D. s* N=6	*D. m* N=5	*D. s* N=6	*D. m* N=5	*D. s* N=6	*D. m* N=6
1	2.62	ND			x	x	6	6	6	5	x	x	x	x	x	x
2	2.74	ND			x	x	6	x	6	x	4	x	6	x	6	x
3	2.84	ND			x	x	x	x	6	x	5	5	6	x	x	x
4	2.94	ND	ND	Ethyl acetate	5	6	6	6	6	5	5	5	6	5	6	6
5	3.39	ND			x	x	6	x	6	5	6	3	x	x	6	5
6	3.45	ND			x	x	x	x	x	x	x	x	4	x	4	6
7	3.49	ND			6	5	x	x	x	x	x	x	x	5	x	x
8	3.69	709	731	Ethyl propionate	6	6	6	6	6	5	6	5	6	5	6	6
9	3.94	732			6	6	6	x	x	x	5	x	5	5	6	6
10	4.08	744			x	6	6	6	6	5	6	5	6	5	4	6
11	4.26	758	772	Ethyl isobutyrate	6	6	x	x	5	x	5	x	6	5	6	4
12	4.48	774	783	Isobutyl acetate	6	6	6	6	6	5	6	x	6	x	6	x
13	4.62	784			x	x	x	x	x	x	6	5	6	3	6	6
14	4.89	801	803	Ethyl butyrate	6	6	x	x	5	x	5	3	x	5	6	6
15	5.05	811	817	Butyl acetate	6	6	6	6	6	5	6	5	6	5	6	5
16	5.14	817			6	6	6	6	6	5	6	x	6	x	6	5
17	5.52	838			6	x	x	x	5	x	6	x	6	5	6	x
18	5.85	856	858	Ethyl isovalerate	6	6	6	6	6	5	6	5	6	5	6	6
19	6.04	865			x	x	x	x	x	x	x	3	x	x	x	x
20	6.16	871			4	4	x	6	x	x	x	x	x	x	6	x
21	6.23	874			x	6	x	x	x	x	x	5	6	5	x	5
22	6.33	879	880	Isoamyl aceate	6	6	6	6	6	5	6	5	6	5	6	6
23	6.52	887			6	x	6	x	6	5	x	x	x	x	6	x
24	6.64	893	892	2-Heptanone	6	6	6	6	6	5	6	5	6	5	6	5
25	6.77	898			6	6	6	6	6	5	6	5	6	5	6	5
26	6.86	902	903	4-Pentenyl acetate	6	6	6	6	6	5	6	x	6	x	6	x
27	7.02	910			5	x	x	x	x	x	6	x	6	x	4	x
28	7.16	917	916	2,5-Dimethylpyrazine	x	6	x	6	6	5	x	5	x	5	6	6
29	7.38	927			x	x	6	6	6	5	x	x	x	x	x	x
30	7.51	933			6	5	6	6	x	x	6	x	6	x	6	6
31	7.66	940			5	6	x	x	x	x	5	5	x	x	6	6
32	7.86	948			6	6	6	6	6	5	6	5	6	5	6	6
33	8.32	967			x	x	6	x	6	x	x	x	6	x	x	x
34	8.48	973	973	Isoamyl propionate	6	6	6	6	6	5	6	x	5	5	6	6
35	8.71	982			6	6	6	6	6	4	6	5	6	5	6	6
36	8.93	990	989	6-Methyl-5-heptene-2-one	6	6	6	6	6	5	6	5	6	5	6	6
37	9.07	995			x	x	x	x	x	x	x	x	x	x	6	6
38	9.11	996	997	Furfuryl acetate	x	x	x	6	6	5	6	5	x	x	x	x
39	9.23	1000	999	Ethyl hexanoate	6	6	x	6	x	5	x	5	x	5	6	6
40	9.36	1006			x	x	x	x	x	x	x	x	x	x	6	6
41	9.40	1008			x	6	6	4	5	5	5	5	x	5	x	6
42	9.69	1020			x	6	6	6	6	5	6	5	5	5	6	6
43	9.98	1032			5	x	6	x	x	x	6	5	6	5	6	6
44	10.40	1049			6	x	5	6	4	x	x	x	4	x	6	x
45	11.59	1093			x	x	6	x	6	x	6	x	5	x	6	x
46	11.98	1107			6	5	6	6	6	5	6	5	6	5	6	6
47	12.19	1116			6	6	6	6	6	5	6	5	6	5	6	6
48	12.46	1127			x	6	6	6	6	5	4	5	6	5	6	6
49	12.48	1128			x	x	x	x	6	x	x	x	x	x	x	x
50	15.05	1227			4	x	x	x	x	x	x	x	x	5	6	x
51	15.91	1261	1261	2-Phenethyl acetate	x	x	6	6	6	5	x	x	x	x	x	x
52	18.28	1358			x	x	5	6	x	x	x	x	x	x	x	x
53	20.61	1456			x	x	x	x	x	x	x	4	x	x	x	x

ND = Not determined

x = Absence of response, either due to the absence or lower amounts (below detection threshold) of a given chemical constituent

* = Identification was based on (1) matching Kovats Indeces between FID and MS, (2) comparing spectra with MS library, (3) confirming KI and biological activity of each synthetic standard

Supplementary Table S2. A compilation of the constituents selected for electrophysiological and yeast chemical analyses assigned with unique compound ID.

Compound ID	Identified constituents	CAS #	Retention time (min.)	Kovats Index
1*			2.62	ND
2			2.74	ND
3			2.84	ND
3a			2.93	ND
4*	Ethyl acetate	141-78-6	2.94	ND
4a	2-Methyl-1-propanol	78-83-1	3.02	ND
5			3.39	ND
6			3.45	ND
7			3.49	ND
8*	Ethyl propionate	105-37-3	3.69	709
9	Isoamyl alcohol	123-51-3	3.94	732
9a	1-Pentanol	71-41-0	3.98	736
9b	2-Methyl-1-butanol	137-32-6	4.02	738
10			4.08	744
11*	Ethyl isobutyrate	97-62-1	4.26	758
12*	Isobutyl acetate	110-19-0	4.48	774
13			4.62	784
14*	Ethyl butyrate	105-54-4	4.89	801
15*	Butyl acetate	123-86-4	5.05	811
16*			5.14	817
17*			5.52	838
18*	Ethyl isovalerate	108-64-5	5.85	856
19			6.04	865
20			6.16	871
21			6.23	874
22*	Isoamyl acetate	123-92-2	6.33	879
22a	2-Methylbutyl acetate	624-41-9	6.38	881
23			6.52	887
24*	2-Heptanone	110-43-0	6.64	893
25*			6.77	898
26*	4-Pentenyl acetate	1576-85-8	6.86	902
27			7.02	910
28*	2,5-Dimethylpyrazine	123-32-0	7.16	917
29			7.38	927
30			7.51	933
31			7.66	940
32*	Citraconic anhydride	616-02-4	7.86	948
33	Benzaldehyde	100-52-7	8.32	967
34*	Isoamyl propionate	105-68-0	8.48	973
35	3-(Methylthio)-1-propanol	505-10-2	8.71	982
35a	Phenol	108-95-2	8.75	983
36*	6-Methyl-5-heptene-2-one	110-93-0	8.93	990
37			9.07	995
38*	Furfuryl acetate	623-17-6	9.11	996
39*	Ethyl hexanoate	123-66-0	9.23	1000
39a	Octanal	124-13-0	9.30	1004
40			9.36	1006
41			9.40	1008
42			9.69	1020
43			9.98	1032
44			10.40	1049
45			11.59	1093
46	Nonanal	124-19-6	11.98	1107
47			12.19	1116
47a	2-Phenylethanol	60-12-8	12.20	1116
48*			12.46	1127
49			12.48	1128
49a	Ethyl octanoate	106-32-1	14.38	1199
49b	Decanal	112-31-2	14.58	1207
50			15.05	1227
51*	2-Phenethyl acetate	103-45-7	15.91	1261
52	Phenethyl propionate	122-70-3	18.28	1358
52a	Ethyl decanoate	110-38-3	19.27	1397
53	Geranylacetone	689-67-8	20.61	1456
53a	Farnesol	4602-84-0	20.88	1468

ND = Not determined

* = Top 10 most effective yeast constituents

Supplementary Figure S1. Annotated chemical profiles (TIC) of six yeast species.

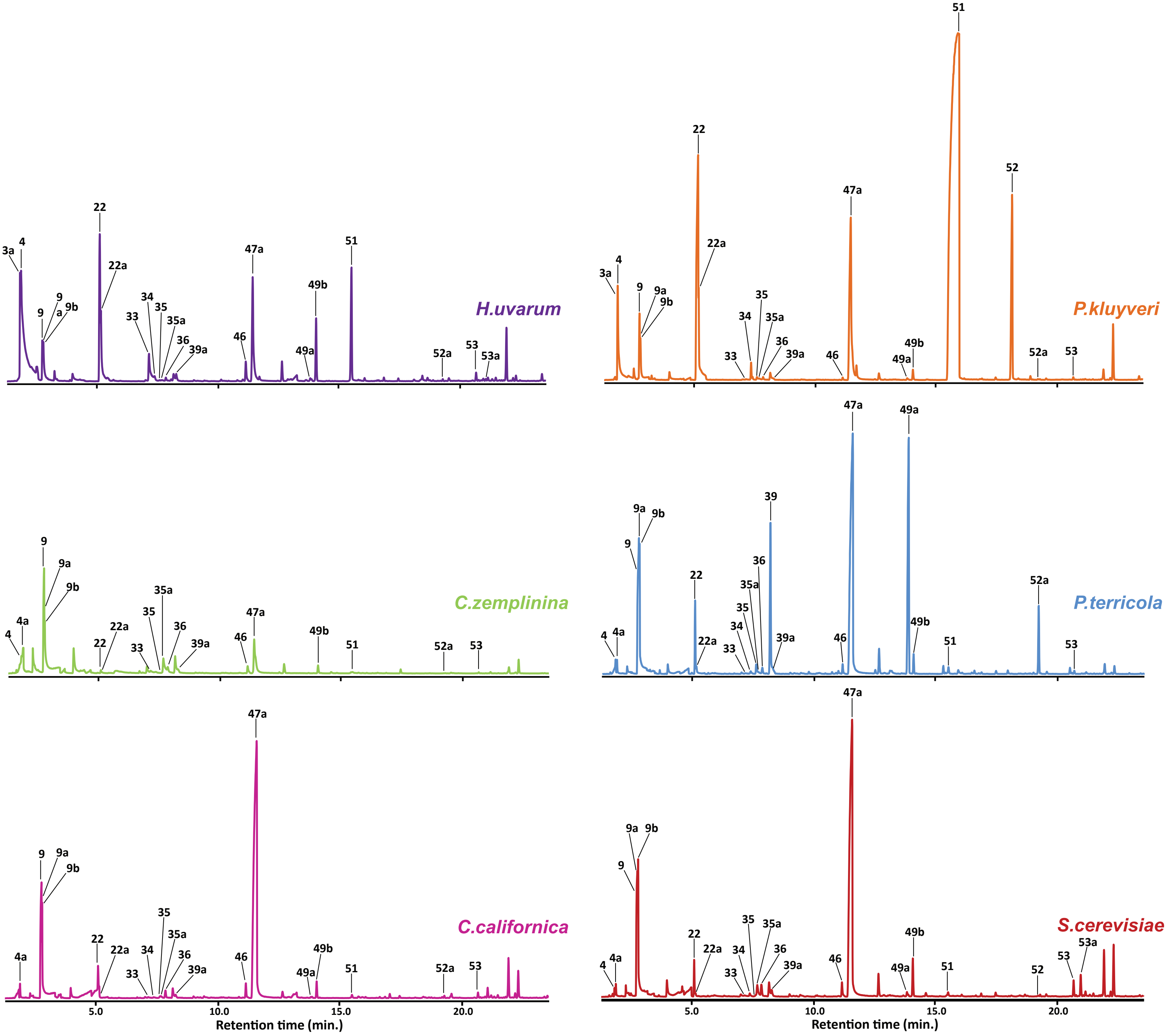

Supplementary Figure S2. Variation in constituent abundances among yeasts as exemplified in the PCA (in Fig. 2). Unique ID of each compound is in parentheses whereas an asterisk (*) indicates that the identification was confirmed with synthetic standards. For each yeast profile, the constituents not part of the initial selection were retrieved or determined not present. Values are percent abundance of the total area (N = 5-7; mean ± SEM).

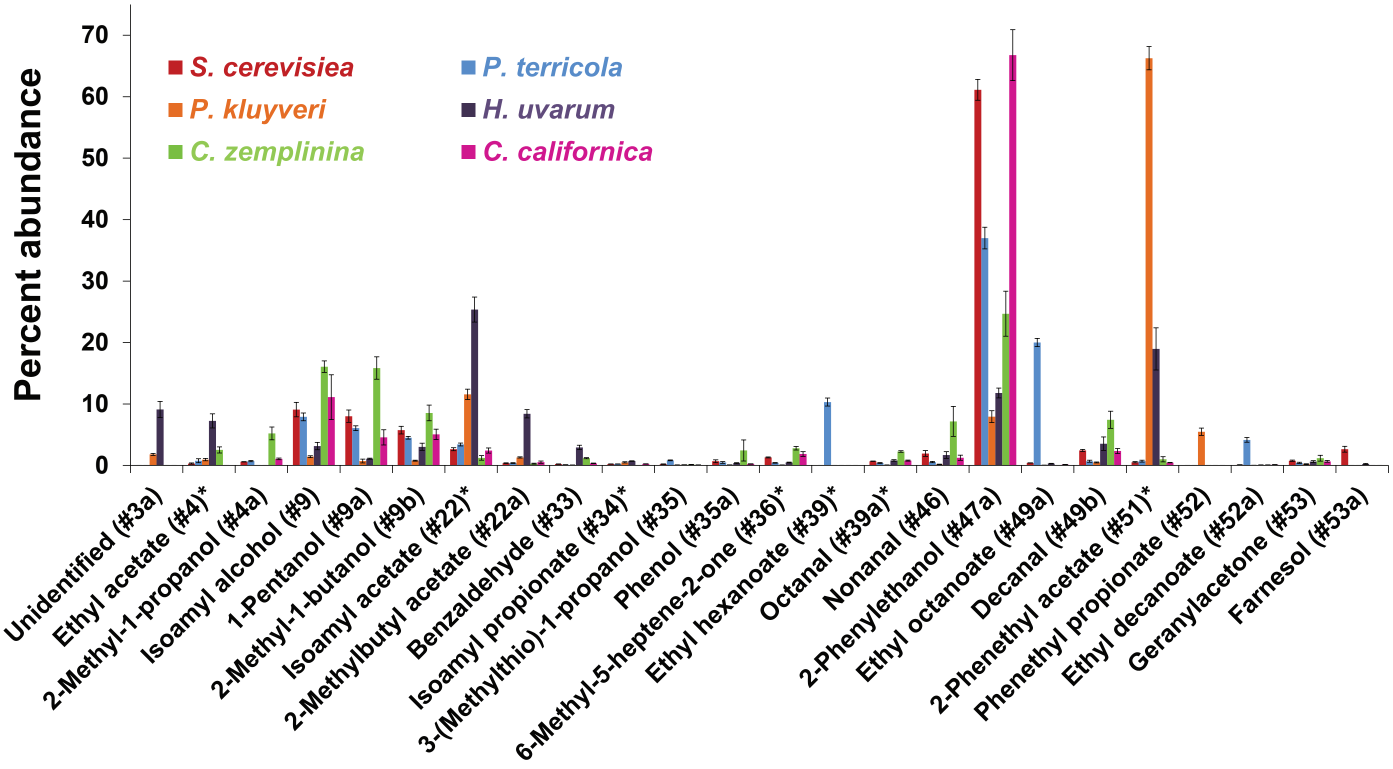

Supplementary Figure S3. Representative GC-EAD profiles. FID traces are represented at the bottom (colored) whereas antennal responses are above [*D. suzukii* (top gray trace) and *D. melanogaster* (middle black)]. Annotated GC-EAD (boxed) profile is from *H. uvarum*.

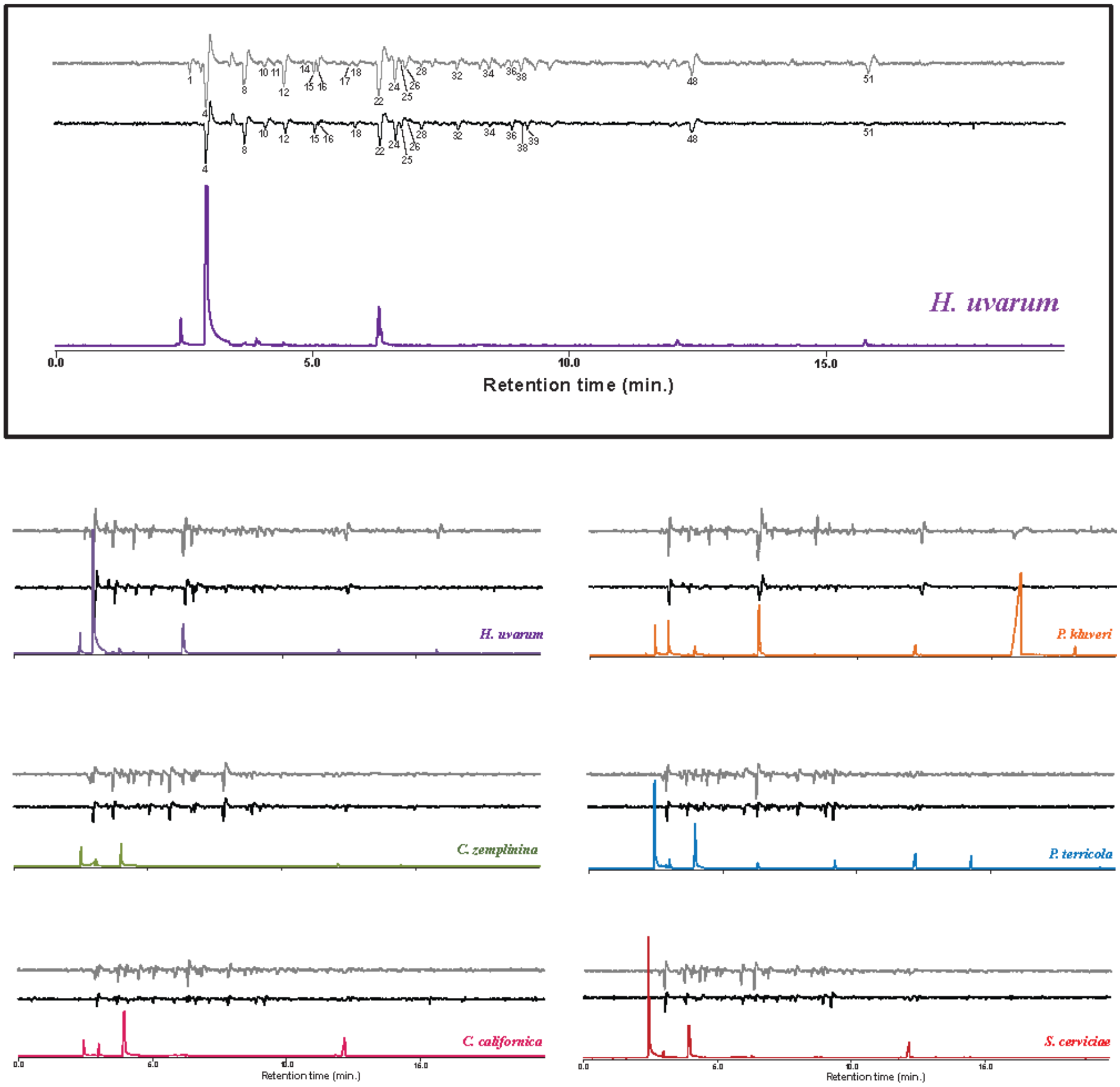

Supplementary Figure S4. Correlation of the relative VOC abundance between yeast spp. and the physiological responses measured from two fly species.

VOCs were produced in varying amounts in each yeast spp. (bottom graph) and elicited antennal responses characterized by different sensitivity and selectivity between *D. melanogaster* (middle) and *D. suzukii* (top). For example, antennal responses to ethyl acetate are comparable, whereas isoamyl acetate and ethyl hexanoate (highlighted with an outline) elicit variable responses between two fly species.

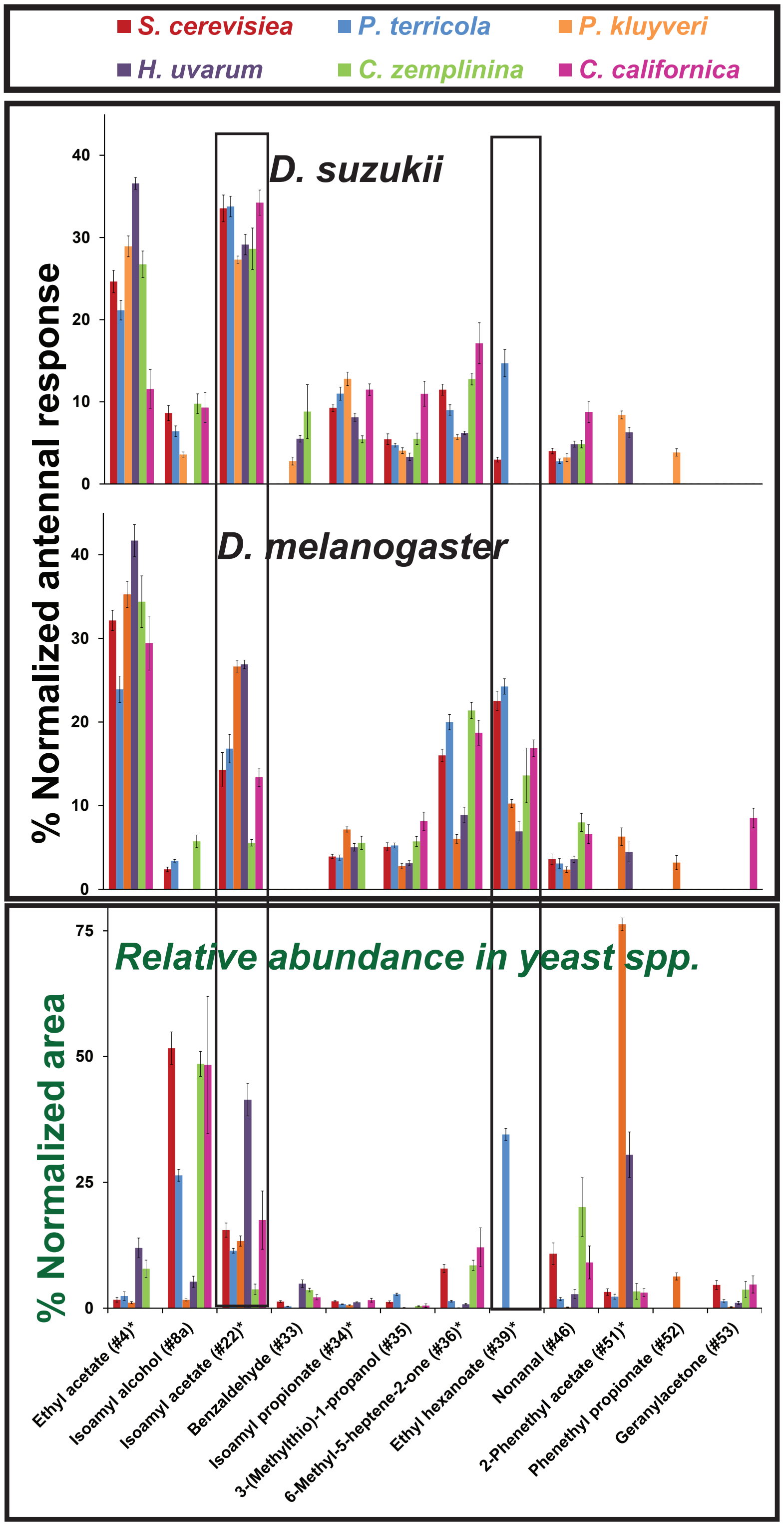

Supplementary Figure S5. Trap and assay details for behavioral experiments. *Left* Trap made by modifying a 1.5 mL centrifuge tube and a 1000 µl pipette tip; *Middle* Binary choice assay used for testing one yeast species against a control (PDB); *Right* Multi-choice assay for determining fly preferences to multiple yeasts spp.

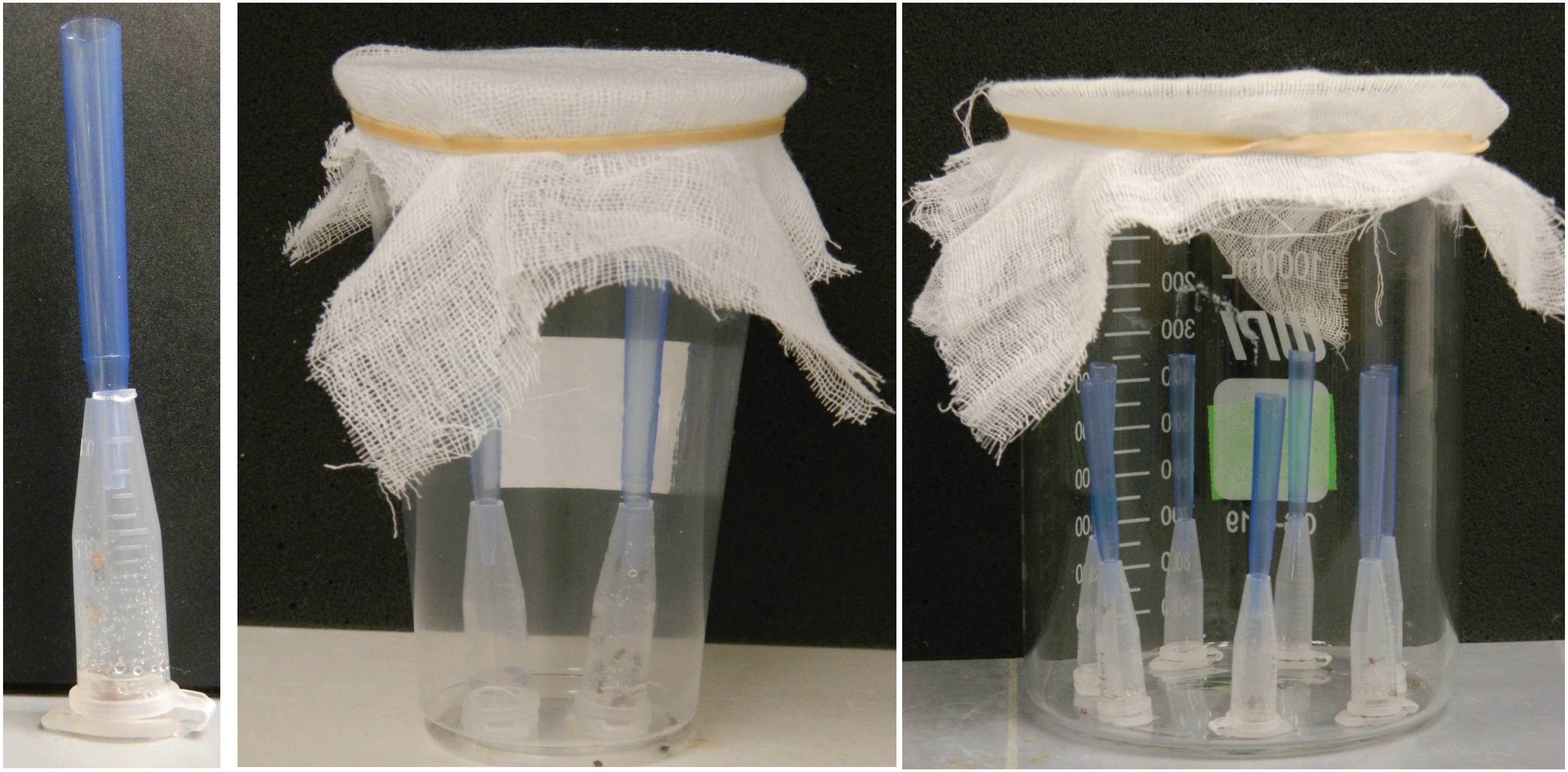

Supplement: Supplementary Information [file srep14059-s1.pdf]
